# An In Vivo Toxicological Study Upon Shallomin, the Active Antimicrobial Constitute of Persian Shallot (Allium hirtifolium, Boiss) Extract

**DOI:** 10.17795/jjnpp-3566

**Published:** 2012-01-04

**Authors:** Mansor Amin, Mohammad Hassan Pipelzadeh, Manijeh Mehdinejad, Iran Rashidi

**Affiliations:** 1 Department of Microbiology, School of Medicine, Infection and Tropical Diseases Research center, Ahvaz Jundishapur University of Medical Sciences, Ahvaz, IR Iran; 2 Department of Pharmacology and Toxicology Research Centre, Ahvaz Jundishapur University of Medical Sciences, Ahvaz, IR Iran; 3 Department of Pathology, School of Medicine, Ahvaz Jundishapur University of Medical Sciences, Ahvaz, IR Iran

**Keywords:** Shallots, Acute Toxicity

## Abstract

**Background:**

Previous studies have shown that shallomin, one of the active constituents of Persian shallot, has a broad range of antimicrobial properties.

**Objectives:**

The safety of shallomin must be established before it can be used in clinical applications. Therefore, the aim of the present study was to evaluate the acute toxic effects of shallomin and to estimate its lethal dose low (LDLo) value.

**Materials and Methods:**

Two series of experiments were performed: In the first series, we used functional testing to assess the acute toxic effects of shallomin on the blood, liver, and kidney and examined histopathological changes in the liver, kidney, lung, and heart, following 7 days of daily intraperitoneal administration of 3 standard doses (10, 20, and 30 µg/g body weight of mice). In the second series, the LDLo value was estimated by determining daily mortality in mice after 7-day administration of escalating doses of shallomin (10 to 240 µg/g body weight of mice).

**Results:**

The results showed that shallomin (at the anticipated in vivo doses), unlike the placebo (ethanol), did not produce any adverse effects on the tested organs. The LDLo value was observed to be 160 µg/g body weight; this value is 8- to 32-times the anticipated in vivo dose that produces antimicrobial effects under in vitro conditions against various pathogenic organisms.

**Conclusions:**

In conclusion, the results of the present study show that shallomin is a relatively safe agent, although its use needs to be carefully monitored. Further in vivo chronic toxicity tests need to be performed to establish the therapeutic potential of shallomin as an antimicrobial agent.

## 1. Background

The Persian shallot (Allium hirtifolium), sometimes mistakenly named Allium ascalonium, belongs to the genus Allium, which includes onion and garlic, and grows wild in the Zagros Mountains, Iran. Previous in vitro studies have shown that the crude extract of the bulb of this plant has antimicrobial properties against a variety of pathogenic bacteria, such as Pseudomonas aeruginosa (1). In vivo investigations showed that 40 mg/mL of the topically applied aqueous extract was useful in the recovery of rats from burn injuries (2). After autoclaving, the crude aqueous extract of shallot, unlike onion and garlic, maintained its antimicrobial activity against both gram-negative and gram-positive pathogenic bacteria (Staphylococcus aureus, Listeria monocytogenes, Bacillus cereus, A. fumicans, Serratia marcescens, Escherichia coli, P. aeruginosa, Camp. species (3), Salmonella typhi, S. paratyphi A, Proteus mirabilis, and Shigella species), with a minimum inhibitory concentration (MIC) ranging from 5 to 20 µg/mL (2). In addition, the crude extract showed fungistatic and fungicidal activity against pathogenic fungi (Microsporum gypseum, Aureobasidium pullulans, Trichophyton mentagrophyte, T. rubrum, Fusarium oxysporum, Saccharomyces cerevisiae, Aspergillus niger, A. flavus, A. fumigatus, and Candida albicans), with a MIC range of 0.15 to 20 mg µg/mL (2). The active compound responsible for the antimicrobial properties is a flavonoid with the general formula C_14_H_8_O_8_ and the proposed name of shallomin (3). Therefore, this ancient plant is a potential source for the treatment of bacterial and fungal infections and has a unique property not commonly found in current anti-infective agents. However, evidence of the safety of any medicinal agent, including those obtained from plants, is required before it can be used in the food industry or in any clinical setting.

## 2. Objectives

The aim of this study was to evaluate the toxicity of shallomin on various body systems from 3 prospective viewpoints: first, by using a range of doses that correlate with effective in vitro concentrations to assess its effect on biochemical parameters related to the blood, liver, and kidney; second, to assess the acute histopathological changes in various organs; and third, to estimate the lethal dose low (LDLo) value, which is the lowest reported concentration that causes death in humans or animals (4). These types of evaluations are believed to be essential when collecting preliminary information on the possible use of this novel agent in clinical practice. This evaluation was thought to be essential in establishing preliminary information on the possible use of shallomin in clinical practice.

## 3. Materials and Methods

### 3.1. Extraction of Shallomin

About 300 g of white shallot bulbs (collected in the spring from the Zagros Mountains; 50 km from Dezful, a city in Southern Iran) was washed thoroughly in water and cut into small pieces by using a kitchen mixer. The pieces were then soaked in 300 mL of distilled water and stirred using a magnetic stirrer for 5 h. Then, the suspension was filtered through Whatman No. 1 filter paper, and the aqueous extract was mixed with ethyl acetate in a 50:50 ratio and stirred for 10 min. The upper organic layer was separated using a separating funnel and centrifuged at 5,000 rpm for 10 min. The ethyl acetate layer was then removed and transferred into a clean flask. This process was repeated 3 times, and the extracts were pooled and dried in a rota-evaporator (Heidolph, Germany) at 50°C. The yield from the extract was weighed and dissolved in ethanol at various concentrations. 

### 3.2. Animal Grouping

A total of 16 groups (n = 10 per group) of male BALB/c mice (body weight, 20 ± 5 g) were used. The groups were housed in separate cages maintained at room temperature, ranging from 25°C to 30°C, with free access to standard laboratory food and tap water. The mice were used in 2 separate series of experiments: in the first series of in vivo experiments, 5 groups (n = 10 per group) were used for the assessment of hematotoxicological, hepatotoxicological, and renal toxicological effects with 3 increasing anticipated doses for the treatment of infections. In the second series designed for assessment of acute toxicity, 11 groups of healthy male mice were used.

### 3.3. Evaluation of Hematotoxicity, Hepatotoxicity, and Renal Toxicity Effects of Shallomin 

In this series of experiments, the first 3 groups (n = 10 per group) were used as the test groups, in which shallomin was administered to the mice at varying doses of 10, 20, or 30 µg/g body weight. The fourth group was used as the placebo group, in which 0.1 mL of ethanol (the solvent) was administered daily via intraperitoneal (IP) injection for 7 consecutive days. The fifth group was used as the non-treated group.

Parameters such as complete blood count (CBC), serum alkaline phosphatase, aspartate aminotranferase (AST), alanine transferase (ALT), creatinine, total bilirubin, direct bilirubin, and blood urea nitrogen (BUN) were measured after 7 days. In addition, the mice were killed, and their lung, liver, kidney, and heart were removed and evaluated for any pathological changes. The dose range for measurement of these parameters ranged from 5 µg/mL to 20 µg/mL (adjusted to body weight in g) and was selected on the basis of previous in vitro concentrations observed to be effective against both fungi and bacteria (2).

### 3.4. Estimation of Lethal Dose Low of Shallomin in Mice

Seven groups of mice (n = 10 per group; average weight, 20 ± 5 g) were administered IP injection of shallomin dissolved in 0.1 mL of 96% ethanol daily for 7 days (10, 40, 80, 120, 160, 200, or 240 µg/g body weight). The mortality rate in each group was assessed daily for 1 week, and the lowest dose that produced mortality during this period was recorded.

### 3.5. Statistical Analysis 

The results were expressed as mean ± standard error values. The significance of the differences between the mean values was assessed using analysis of variance, followed by Duncan’s test for multiple comparisons among the different groups. A P value <0.05 was considered significant.

## 4. Results

CBC analysis of the ethanol-treated group showed a significant reduction in platelet and red blood cell (RBC) counts, in comparison with those in both the non-treated control and shallomin-treated groups; this suggested that ethanol causes significantly acute suppression of the bone marrow. Shallomin, at the doses used, reversed the platelet-reducing effect of ethanol. However, the ethanol-treated group showed significant reduction in hematocrit and serum hemoglobin levels, which were partially reversed in the shallomin-treated group (Table 1). Furthermore, the white blood cell (WBC) counts increased in the ethanol-treated group; however, shallomin had no effect on the ethanol-induced increase in the WBC count (Table 1).

**Table 1:** Mean ± SEM SD values of various experimental parameters measured after 1 week of daily administration of low (10 μg/g), intermediate (20 μg/g), and high dose (30 μg/g) of shallomin in the shallomin-treated groups, in comparison with that in the ethanol- (placebo) and non-treated groups.

	PLT ^C^	HCT ^C^	HGB ^C^	RBC ^C^	WBC ^C^	ALK ^C^	ALT ^C^	AST ^C^	DBil ^C^	TBil ^C^	Cr ^C^	BUN ^C^
Low dose 10 µg/g	849.4 ± 54.8	40.6 ± 0.69	13.5 ± 0.22	7a ± 0.1	8.6a ± 2.8	515 ± 47	2.66 ± 0.3	1.6 ± 0.11	0.27 ± 0.01	0.61 ± 0.01	0.51 ± 0.03	81 8 ± 0.9
Intermediate dose 20 µg/g	835.6 ± 30	42.4 ± 1.45	13.3 ± 0.43	7.2 ± 0.2	9.9 ± 0.7	482 ± 29	3.3 ± 0.5	1.4 ± 0.26	0.26 ± 0.01	0.56 ± 0.02	0.5 ± 0.03	8 ± 0.8
High dose 30 µg/g	856.5 ± 29	38.1 ± 0.7	12.7 ± 0.24	6.9 ± 0.2	9.6 ± 0.8	424 ± 29	2.2 ± 0.5	1.4 ± 0.42	0.24 ± 0.01	0.61 ± 0.02	0.46 ± 0.03	10 ± 0.7
Placebo- ethanol	560.6 ± 48	37.7^b^ ± 0.23	12.1^b^ ± 0.21	7.04 ± 0.21	9.44 ± 0.35	466 ± 29	3 ± 0.16	1.1 ± 0.21	0.23 ± 0.01	0.48^b^± 0.02	0.55^b^ ± 0.03	9.3^b^± 0.35
Negative control	765.9^a^ ± 43	45.7^a^ ± 1.1	15.4^b^ ± 0.3	8a ± 0.2	7.3 ± 0.8	462 ± 43	6.9 a ± 0.4	1.55 ± 0.2	0.27 ± 0.01	0.77a ± 0.02	0.82a ± 0.04	8.7 ± 0.6

^a^P < 0.001, compared to the placebo group (placebo effect).

^b^P < 0.01, compared to the shallomin-treated groups.

^c^Abbreviations: ALK: Alkaline Phosphate; BUN: Blood Urea Nitrogen; Cr: Creatinine; DBil: Direct bilirubin; HGB: Hemoglobin; PLT: Platelet; PT: Prothrombin Time; RBC: Red Blood Cells; TBil: Total Bilirubin; WBC: White Blood Cells.

With respect to hepatic toxicity, ethanol alone caused significant reduction in ALT, AST, and total bilirubin levels. These changes were not observed following shallomin treatment; however, the total serum bilirubin level was within the normal range after shallomin treatment. However, the direct bilirubin level (conjugated form of bilirubin) in the ethanol- and shallomin-treated groups did not differ from that in the non-treated group. The serum creatinine level decreased significantly in the ethanol-treated group, but did not change in the shallomin-treated groups, in comparison with that in the control group. In addition, the serum BUN level increased in the ethanol-treated group, but it was within the normal range in the shallomin-treated group (Table 1).

### 4.1. Histopathological Findings after Shallomin Administration 

No remarkable histopathological changes were observed in the lung, liver, heart, or kidneys of the different treatment groups. 

### 4.2. Lethality of Shallomin in Mice 

After 7 days of daily escalating doses of shallomin, the percentage of mice that died was found to proportionally increase in a dose-dependent manner (Table 2). Among the selected groups, the first incidence of death was observed on the second day of administration with a dose of 160 µg of shallomin per gram of body weight (Table 2).

**Table 2:** Cumulative number of deceased mice per group, following 1 week of daily intraperitoneal administration of escalating doses (10 to 240 μg/g body weight) of shallomin dissolved in 0.1 mL ethanol. The lowest dose that caused mortality was observed with 160 μg body weight on the day following shallomin administration of shallomin administration.

Day 7	Day 6	Day 5	Day 4	Day 3	Day 2	Day 1	Group-Dose/Body Weight, μg/g
0	0	0	0	0	0	0	0
0	0	0	0	0	0	0	10
0	0	0	0	0	0	0	20
0	0	0	0	0	0	0	40
0	0	0	0	0	0	0	60
0	0	0	0	0	0	0	80
0	0	0	0	0	0	0	100
0	0	0	0	0	0	0	120
3	3	3	3	3	2	0	160
6	7	6	5	4	3	0	200
7	7	6	6	6	5	0	240

## 5. Discussion

Assessment of toxicity of an agent, regardless of its source, is an integral part for its future use as a therapeutic agent. Previous studies (1, 5-7) had evaluated the antibacterial properties of raw shallot extract; however, none of the studies had attempted to isolate and test the active ingredient responsible for this property. To complement our previous in vitro studies on shallomin, we evaluated the toxic effects of shallomin (the active antimicrobial constituent of shallot) in mice. One of the main limitations of our present study was the solubility of shallomin, which is soluble in ether, methanol, and ethanol. We selected ethanol because it is considered the safest solvent. However, acute biochemical toxicity was observed in this group; this highlighted the significant toxic effects of this agent. With respect to the assessed parameters, variable results in the ethanol-treated group primarily affected the blood, kidney, and hepatic systems of the mice. In addition, the number of platelets decreased in the ethanol-treated group, but not in the shallomin-treated groups; this finding suggested that shallomin has a protective effect against the platelet-reducing effects of ethanol. These toxic effects seem to be related to the bone marrow suppression property of ethanol in acute doses. The nature of such suppressive effects on the bone marrow needs to be assessed further in future studies. In addition, ethanol significantly reduced the RBC count and reduced the hemoglobin and hematocrit levels. The shallomin-treated groups showed similar effects, suggesting that these observed effects on the hemopoietic system were mainly caused by ethanol and that shallomin had no direct toxic effects on this system.

Previous studies reported isolation of various constituents identified as flavonoids, saponins, and lectins from shallomin (8, 9). However, none of these compounds possessed antibacterial properties. Ascalin, a unique specific anti-fungal agent without antibacterial activity (10), was isolated and identified by Wang and Nag in 2001. This peptide, derived from another species of shallot (A. ascalonicum), has a molecular weight of 9.5 kDa and possesses an N-terminal sequence of YQCGQGG.

When the renal system parameters were determined, the serum creatinine level was observed to have decreased to a similar degree in both ethanol- and shallomin-treated groups, suggesting that the decreased values were related to the effects of ethanol. However, serum BUN levels had increased in the ethanol-treated group and had decreased in the shallomin-treated group; this suggested that shallomin had no adverse effects on BUN. In fact, shallomin reversed the ethanol-induced upregulation of BUN. These effects may be attributable to the renal protective effects of shallomin. However, the specific protective effects of shallomin on the renal system need to be studied further.

No changes in alkaline phosphatase, total bilirubin, or direct bilirubin were observed in the ethanol-treated and shallomin-treated groups. These findings suggest that, at the doses tested, both ethanol and shallomin had no adverse effects on hepatic function. In fact, ALT levels, a marker for acute toxicity in hepatic cells, had decreased in both groups.

The doses used in this study were selected on the basis of concentrations found effective under in vitro conditions against most pathogenic fungi and bacteria. Previous in vitro studies showed that the aqueous extract of crude shallot had both antibacterial and antifungal effects, comparable to conventionally used antibiotics, with an antifungal MIC range of 0.15 to 20 mgµg/mL and an antibacterial MIC range of 5 to 20 mgµg/mL (2). Furthermore, in vivo studies using a burn model found an effective topical dose of 4 mg/mL. These concentrations are within the normal range of activity of most antibiotics. However, the LDLo value of shallomin was found to be 160 µg per gram of body weight; this value, in terms of the ratio of lethality dose to effective concentration, is between 8- and 32-times the in vivo dose anticipated to produce antimicrobial effects against various pathogenic organisms. These findings suggest that shallomin is a relatively safe agent; however, the in vivo and clinical uses of shallomin need to be carefully monitored. Further chronic toxicity tests and clinical experiments need to be performed to establish the therapeutic potential of shallomin as an antimicrobial agent.
